# Lecanemab in patients with early Alzheimer’s disease: detailed results on biomarker, cognitive, and clinical effects from the randomized and open-label extension of the phase 2 proof-of-concept study

**DOI:** 10.1186/s13195-022-01124-2

**Published:** 2022-12-21

**Authors:** Eric McDade, Jeffrey L. Cummings, Shobha Dhadda, Chad J. Swanson, Larisa Reyderman, Michio Kanekiyo, Akihiko Koyama, Michael Irizarry, Lynn D. Kramer, Randall J. Bateman

**Affiliations:** 1 The DIAN–TU, Department of Neurology, Washington University School of Medicine, St. Louis, MO USA; 2 Chambers-Grundy Center for Transformative Neuroscience, Quirk Brain Health and Biomarker Laboratory, Department of Brain Health, School of Integrated Health Sciences, University of Nevada Las Vegas, Las Vegas, NV USA; 3 Eisai Inc., Nutley, NJ USA

## Abstract

**Background:**

Lecanemab, a humanized IgG1 monoclonal antibody that targets soluble aggregated Aβ species (protofibrils), has demonstrated robust brain fibrillar amyloid reduction and slowing of clinical decline in early AD. The objective of this analysis is to report results from study 201 blinded period (core), the open-label extension (OLE), and gap period (between core and OLE) supporting the effectiveness of lecanemab.

**Methods:**

The lecanemab study 201 core was a double-blind, randomized, placebo-controlled study of 856 patients randomized to one of five dose regimens or placebo. An OLE of study 201 was initiated to allow patients to receive open-label lecanemab 10mg/kg biweekly for up to 24 months, with an intervening off-treatment period (gap period) ranging from 9 to 59 months (mean 24 months).

**Results:**

At 12 and 18 months of treatment in the core, lecanemab 10 mg/kg biweekly demonstrated dose-dependent reductions of brain amyloid measured PET and corresponding changes in plasma biomarkers and slowing of cognitive decline. The rates of clinical progression during the gap were similar in lecanemab and placebo subjects, with clinical treatment differences maintained after discontinued dosing over an average of 24 months in the gap period. During the gap, plasma Aβ42/40 ratio and p-tau181 levels began to return towards pre-randomization levels more quickly than amyloid PET. At OLE baseline, treatment differences vs placebo at 18 months in the randomized period were maintained across 3 clinical assessments. In the OLE, lecanemab 10 mg/kg biweekly treatment produced dose-dependent reductions in amyloid PET SUVr, improvements in plasma Aβ42/40 ratio, and reductions in plasma p-tau181.

**Conclusions:**

Lecanemab treatment resulted in significant reduction in amyloid plaques and a slowing of clinical decline. Data indicate that rapid and pronounced amyloid reduction correlates with clinical benefit and potential disease-modifying effects, as well as the potential to use plasma biomarkers to monitor for lecanemab treatment effects.

**Trial registration:**

ClinicalTrials.gov NCT01767311.

**Supplementary Information:**

The online version contains supplementary material available at 10.1186/s13195-022-01124-2.

## Introduction

Alzheimer’s disease (AD) is a major health problem of aging with tremendous burden on healthcare systems globally [1–4]. Although there are symptomatic therapies approved, they provide modest clinical benefit and have no impact on disease progression. One anti-amyloid antibody (aducanumab) has accelerated approval from the FDA; it does not fulfill the unmet need for AD therapy. Therefore, disease-modifying therapies are critically needed to improve the lives of those with AD and to decrease the global burden of the disease.

Amyloid beta (Aβ) pathology has been identified as a target for intervention based on the evidence that it likely plays an important role in the development and progression of the disease. However, nearly all symptomatic AD trials targeting Aβ-pathology have been unsuccessful in demonstrating a clinical benefit [5–7]. Only recently have specific therapies that effectively target Aβ-pathology been developed and include those that substantially reduce aggregated Aβ plaques, decrease soluble Aβ, or reduce the production of aggregation prone Aβ species [8–11]. Although it remains uncertain which form of aggregated Aβ is likely to be most pathologic, soluble aggregates (e.g., large, soluble protofibrils) are a rational target based on evidence that these may be the most toxic forms [12–14]. Lecanemab (BAN2401) is a humanized IgG1 monoclonal antibody that binds to large soluble Aβ aggregates (protofibrils) with high selectivity over monomers (>1000-fold) and insoluble fibrils (at least 10-15-fold) [15–18].

In a phase 2 randomized study (study 201 core), lecanemab treatment led to a robust, dose-dependent reduction in brain amyloid, slower decline on clinical outcome measures, and directionally consistent biomarker changes at 18 months [19]. An open-label extension (OLE) was initiated following analysis of core data, resulting in a gap period (no study drug treatment) between the end of the core and the beginning of the OLE. Here, we report detailed results from study 201 core, gap period, and OLE phase supporting the effectiveness of lecanemab, including plasma biomarker outcomes, clinical efficacy, and exposure response (ER) data as well as correlations among positron emission tomography (PET) measures, plasma biomarker assessments, and clinical efficacy evaluations. This study addressed efficacy only and does not present data on safety or tolerability which are addressed elsewhere [19].

## Patients and methods

### Study design

The lecanemab 201 trial (ClinicalTrials.gov Identifier: NCT01767311) was a multinational, multicenter, double-blind, placebo-controlled, parallel-group study (core) employing Bayesian design with response-adaptive randomization with an OLE (Figure S1). Methods and primary results for the study 201 core phase have been published [19]. Briefly, at entry into the core study, subjects were required to have early AD (amyloid positive) with global Clinical Dementia Rating (CDR) global score of 0.5 or 1. Subjects were randomized to either placebo or one of 5 active arms of lecanemab (2.5 mg/kg biweekly, 5 mg/kg monthly, 5 mg/kg biweekly, 10 mg/kg monthly, 10 mg/kg biweekly) without titration. Treatment duration of the study was 18 months with a 3-month follow-up and a target enrollment of approximately 800 subjects. The primary outcome was based on a Bayesian analysis at 12 months; the study continued per protocol with no unblinding to month 18. To maintain the blind during the double-blind portion of the trial, all subjects received biweekly infusions of either placebo or lecanemab.

The 201 OLE was initiated following analysis of the core phase 2b study 201 to allow subjects to receive open-label lecanemab 10 mg/kg biweekly (initiated without titration) for up to 24 months to assess long-term safety and tolerability. All subjects who fulfilled OLE inclusion/exclusion criteria and entered the OLE received 10 mg/kg biweekly during the OLE period. There was a gap period between the end of the study 201 core and OLE baseline when no treatment was provided. The gap period lasted for an average of 24 months (range 9–59 months) for all subjects who entered the OLE. Core treatment assignments remained blinded to study sites and study participants throughout the OLE.

### Study assessments

Study assessments for the study 201 core and OLE included the Alzheimer’s Disease Composite Score (ADCOMS); Clinical Dementia Rating Sum-of-Boxes (CDR-SB); Alzheimer’s Disease Assessment Scale-Cognitive Subscale (ADAS-Cog14); changes in plasma biomarkers; and brain amyloid by PET Standardized Uptake Value ratio (SUVr) (in an optional substudy of consenting participants). The core amyloid PET substudy assessed baseline, 12-month, and 18-month SUVr with florbetapir; the OLE amyloid PET substudy assessments were at baseline, 3 or 6 months, 12 and 24 months. Plasma samples were collected at the same timepoints as the PET studies. Imaging (PET with florbetapir tracer) and plasma (amyloid-β (Aβ)42/40 ratio, phospho-tau (p-tau)181) biomarkers were evaluated. The amyloid PET SUVr normalized to whole cerebellum mask, measured using [18]F florbetapir as a PET ligand, was used to determine brain amyloid levels [20]. Plasma concentrations of Aβ42 and Aβ40 were measured using the immunoprecipitation/liquid chromatography-mass spectrometry/mass spectrometry (IP/LC-MS/MS) technology platform (Precivity AD assay, C2N), and the ratio of plasma Aβ42/40 was calculated from the output. Plasma concentrations of p-tau181 were measured using a commercially validated single molecule array (Simoa) assay developed by Quanterix. Information related to drug interference with plasma Aβ42/40 and p-tau181 assays can be found in the supplement.

### Exposure response (ER) analyses for PET SUVr

The relationship between serum lecanemab concentration and the PET SUVr reduction time course in study 201 core and OLE phase was characterized by an indirect response model for the lecanemab concentration inducing the reduction of brain amyloid. Estimated parameters included baseline SUVr(t) at time=0 (BSUVr0), indirect response rate constant parameters (Kin and Kout [estimated as Kout = Kin/SUVr(0)]), maximum drug effect (Emax), and lecanemab concentration resulting in half of the maximum drug effect (EC50).

$$\frac{dSUVr}{dt}= Kin- SUVr(t)\ast Kout\ast \left[1+\frac{Emax\ast BAN2401\ Conc.}{EC50+ BAN2401\ Conc.}\right]$$

Inter-individual variability was estimated for baseline and Emax and could not be estimated for Kin and EC50. Residual variability was modeled using a proportional model. Covariates tested were sex (women vs men), age, neutralizing anti-drug antibodies (ADA) (positive vs negative), and apolipoprotein E4 (ApoE4) carrier status (positive vs negative only for Emax).

### Exposure response analyses for efficacy

Longitudinal pharmacokinetic/pharmacodynamic (PK/PD) models were developed for efficacy endpoints (ADCOMS, CDR-SB, and ADAS-Cog) using data from the phase 2 study. A disease progression model was developed using data from placebo-treated subjects. Effect of model-predicted lecanemab exposure (maximum concentration at steady state [Css,max] and average concentration at steady state [Css,av]) on disease progression was investigated from data in all subjects as follows:

$$\textrm{EFF}-\textrm{INT}+\textrm{SLP}\ast \left(1-\textrm{DESLOPE}\ast \left(\textrm{lecanemab}\ \textrm{Exposure}\right)\right)\ast \textrm{Time}$$

where EFF, INT, SLP and DESLOPE are clinical scores of efficacy endpoints at each assessment time (EFF), baseline clinical score (INT), disease progression rate (SLP), and lecanemab effect on disease progression rate (DESLOPE), respectively.

Evaluated covariates were age, sex, ApoE4 carrier status (positive or negative), ongoing treatment with acetylcholinesterase inhibitors (AChEIs) and/or memantine (yes or no), and clinical subgroup (mild cognitive impairment (MCI) due to AD or mild AD dementia).

### Relationship between PET SUVr and clinical efficacy

Relationships for change from baseline (CFB) of SUVr whole cerebellum (SUVrWC) versus CFB of clinical endpoints (ADCOMS, CDR-SB, and ADAS-Cog) at 12 and 18 months were explored in a subset of subjects who had post-baseline assessments for both endpoints. The relationships were modeled using a nonlinear effects model. A linear model (CFB of Clinical Endpoint = Intercept + Slope * CFB of PET SUVr) was explored for key clinical endpoints (ADCOMS, CDR-SB, and ADAS-Cog). For all endpoints, inter-individual variability (IIV) was estimated for intercept, and residual variability was modeled using an additive model. Effects of age, sex, ApoE4 carrier status, ongoing treatment with AChEIs and/or memantine (yes or no), and clinical subgroup (MCI due to AD or mild AD dementia) were evaluated as covariates. ER analyses for PET SUV efficacy were performed using nonlinear mixed-effect modeling in NONMEM® version 7.3. Where applicable, the final ER models were evaluated for performance using graphical assessment, non-parametric bootstrapping, and visual predictive checks.

### Statistics

Statistical analyses for study 201 core have been previously published (Swanson 2021). In the study 201 OLE analyses, the focus is on de novo subjects (core placebo-treated) and those on 10mg/kg biweekly from beginning of study (delay start and early start design on most effective dosing regimen). Analyses were conducted in 2 cohorts based on their treatment allocation during study 201 core: (1) subjects who received prior placebo and (2) subjects with prior lecanemab 10 mg/kg biweekly. The change from OLE baseline in change in clinical endpoints (CDR-SB, ADCOMS, ADAS-Cog14) were analyzed using the mixed model repeated measures (MMRM) approach, incorporating key covariates into the model, ApoE4 status, clinical subgroup (MCI due to AD or mild AD dementia), ongoing treatment with AChEIs and/or memantine, and baseline value. Analyses of amyloid PET (SUVr and Centiloid approaches), plasma Aβ42/40, and plasma p-tau181 were also performed. Plasma biomarkers were measured for subjects with available samples. The correlations among the 3 biomarkers and their correlations with clinical endpoints were evaluated using population-level and subject-level correlation analysis. The OLE protocol was drafted and initiated after completion of the core study.

## Results

### Subjects

A total of 856 subjects were randomized into the study to receive either placebo (247 subjects) or lecanemab (609 subjects). Of these, 552 subjects (177 placebo, 375 lecanemab) completed study 201 core. Of the 856 subjects randomized in study 201 core, 180 subjects entered the OLE phase to receive lecanemab 10 mg/kg biweekly, with 45, 38, and 97 having received placebo, lecanemab 10 mg/kg biweekly, or a different lecanemab dose in the core, respectively. The PET substudy included 315 subjects in core and 91 in OLE (22, 21, and 48 having received placebo, lecanemab 10 mg/kg biweekly, or a different lecanemab dose in the core, respectively). A summary of baseline characteristic for subjects in study 201 core and OLE are shown in Table 1.

**Table 1:** Baseline characteristics

Category		CORE						OLE		
			Lecanemab					Lecanemab		
		Placebo (*N*=238)	2.5 mg/kg biweekly (***N***=52)	5 mg/kg monthly (***N***=48)	5 mg/kg biweekly (***N***=89)	10 mg/kg monthly (***N***=246)	10 mg/kg biweekly (***N***=152)	Prior core placebo (***N***=42)	Prior core 10 mg/kg biweekly (***N***=37)	10 mg/kg biweekly (***N***=180)
Age (year)^a^	Mean (SD)	71.1 (8.9)	70.5 (8.3)	70.4 (7.5)	70.6 (7.4)	71.3 (7.5)	72.6 (8.8)	71.8 (8.2)	76.9 (7.0)	74.0 (7.7)
Sex, *n* (%)	Female	137 (57.6)	26 (50.0)	24 (50.0)	48 (53.9)	110 (44.7)	64 (42.1)	21 (50.0)	18 (48.6)	87 (48.3)
Region, *n* (%)	North America	195 (81.9)	47 (90.4)	41 (85.4)	70 (78.7)	215 (87.4)	135 (88.8)	30 (71.4)	32 (86.5)	139 (77.2)
	Western Europe	28 (11.8)	4 (7.7)	6 (12.5)	7 (7.9)	15 (6.1)	10 (6.6)	3 (7.1)	2 (5.4)	12 (6.7)
	Asia Pacific	15 (6.3)	1 (1.9)	1 (2.1)	12 (13.5)	16 (6.5)	7 (4.6)	9 (21.4)	3 (8.1)	29 (16.1)
Amyloid PET SUVr*	Mean (SD)	1.4 (0.2)	1.4 (0.1)	1.4 (0.2)	1.4 (0.1)	1.4 (0.2)	1.4 (0.2)	1.4 (0.2)	1.1 (0.1)	1.2 (0.2)
Amyloid Centiloids*	Mean (SD)	84.8 (37.4)	87.7 (26.4)	89.4 (39.7)	84.9 (28.0)	90.3 (41.5)	78.0 (38.0)	77.2 (42.0)	8.6 (30.9)	44.5 (43.9)
CDR-Global, *n* (%)	0.5	200 (84.0)	44 (84.6)	40 (83.3)	77 (86.5)	210 (85.4)	133 (87.5)	19 (45.2)	19 (51.4)	80 (44.4)
	1	38 (16.0)	8 (15.4)	8 (16.7)	12 (13.5)	13 (14.6)	19 (12.5)	18 (42.9)	11 (29.7)	68 (37.8)
CDR-SB	Mean (SD)	2.9 (1.5)	3.0 (1.6)	2.9 (1.4)	3.0 (1.3)	2.9 (1.3)	3.0 (1.4)	4.7 (3.2)	5.0 (3.7)	5.3 (3.5)
*ApoE4* status, *n* (%)	Carrier	169 (71.0)	38 (73.1)	37 (77.1)	81 (91.0)	218 (88.6)	46 (30.3)	30 (71.4)	3 (8.1)	125 (69.4)
	Heterozygous	129 (54.2)	33 (63.5)	26 (54.2)	67 (75.3)	160 (65.0)	38 (25.0)	4 (9.5)	0	97 (53.9)
	Homozygous	40 (16.8)	5 (9.6)	11 (22.9)	14 (15.7)	58 (23.6)	8 (5.3)	26 (61.9)	3 (8.1)	28 (15.6)
	Noncarrier	69 (29.0)	14 (26.9)	11 (22.9)	8 (9.0)	28 (11.4)	106 (69.7)	12 (28.6)	34 (91.9)	55 (30.6)
Disease stage, *n* (%)	MCI due to AD	154 (64.7)	34 (65.4)	33 (68.8)	52 (58.4)	166 (67.5)	90 (59.2)	27 (64.3)	22 (59.5)	110 (61.1)
	Mild AD	84 (35.3)	18 (34.6)	15 (31.3)	37 (41.6)	80 (32.5)	62 (40.8)	15 (35.7)	15 (40.5)	70 (38.9)
ADCOMS	Mean (SD)	0.4 (0.2)	0.4 (0.2)	0.4 (0.2)	0.4 (0.2)	0.4 (0.2)	0.4 (0.2)	0.6 (0.3)	0.6 (0.4)	0.7 (0.4)
ADAS-cog14	Mean (SD)	22.6 (7.7)	22.7 (8.1)	22.9 (7.7)	22.8 (6.7)	21.9 (7.3)	22.1 (7.7)	33.4 (13.5)	32.5 (13.8)	35.1 (14.0)
MMSE	Mean (SD)	26.0 (2.3)	25.7 (2.5)	25.3 (2.6)	25.6 (2.3)	25.7 (2.4)	25.6 (2.4)	21.5 (6.3)	21.2 (6.0)	20.7 (6.6)

*AChEIs* acetylcholinesterase inhibitor, *AD* Alzheimer’s disease, *ApoE4* apolipoprotein E4, *CDR* Clinical Dementia Rating, *MCI* mild cognitive impairment, *OLE* open-label extension, *SD* standard deviation, *ADAS-Cog14* Alzheimer Disease Assessment Scale - Cognitive Subscale with 14 tasks, *ADCOMS* Alzheimer’s Disease Composite Score, *CDR-SB* Clinical Dementia Rating Sum-of-Boxes, *Max* maximum, *Min* minimum, *MMSE* Mini-Mental State Examination. a: Age was calculated at date of informed consent. Percentages are based on total number of subjects with non-missing values in relevant treatment group; for OLE, age is calculated at Date of Informed Consent for OLE Phase. MCI due to AD: (a) meet NIA-AA core clinical criteria for MCI due to AD; (b) CDR score of 0.5 and Memory box score of 0.5 or greater at screening and baseline; (c) Report of a history of subjective memory decline w/ gradual onset and slow progression over last year before screen (verified by informant). Mild AD: (a) meet NIA-AA core clinical criteria for probable AD dementia; (b) CDR score of 0.5 to 1.0 and Memory box score of 0.5 or greater at screening and baseline

* The number of subjects in amyloid substudy was 315 in core and 91 in OLE (core: placebo=99; 2.5 mg/kg biweekly=28; 5 mg/kg monthly=28; 5 mg/kg biweekly=27; 10 mg/kg monthly=89; 10 mg/kg biweekly=44; OLE: prior core placebo=27; prior core placebo=22, prior core 10BW==21, total=91. 42 out of 45 subjects in prior core placebo group and 37 out of 38 subjects in prior core 10 mg/kg biweekly group are included in OLE Full Analysis Set and summarized here

### Core phase

**Fig. 1:**
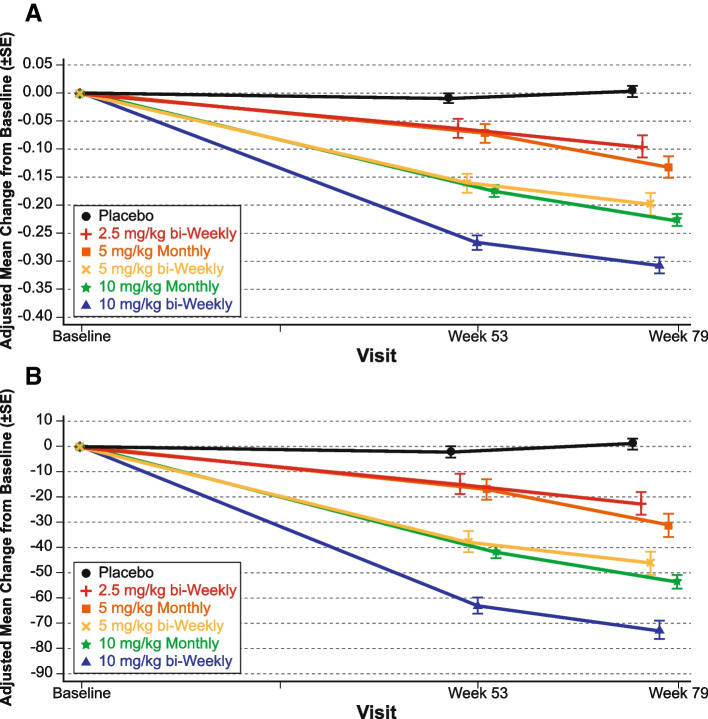
Results for **A** amyloid PET SUVr and **B** Centiloid scale assessments from study 201 core

The results from the primary analysis of the study 201 core phase, including clinical efficacy and biomarkers, have previously been published [19]. Briefly, lecanemab treatment resulted in reduction in brain amyloid accompanied by a consistent reduction of clinical decline across several clinical and biomarker endpoints. The least squares (LS) mean change from baseline in brain amyloid levels as measured by amyloid PET SUVr and in Centiloid scales are shown in Fig. 1 (Centiloid data have not been previously published). Lecanemab demonstrated a dose-dependent and time-dependent brain amyloid reduction across all doses versus placebo. Overall, 65% of subjects at 12 months and 81% of subjects at 18 months converted from amyloid positive to amyloid negative by visual read.

**Fig. 2:**
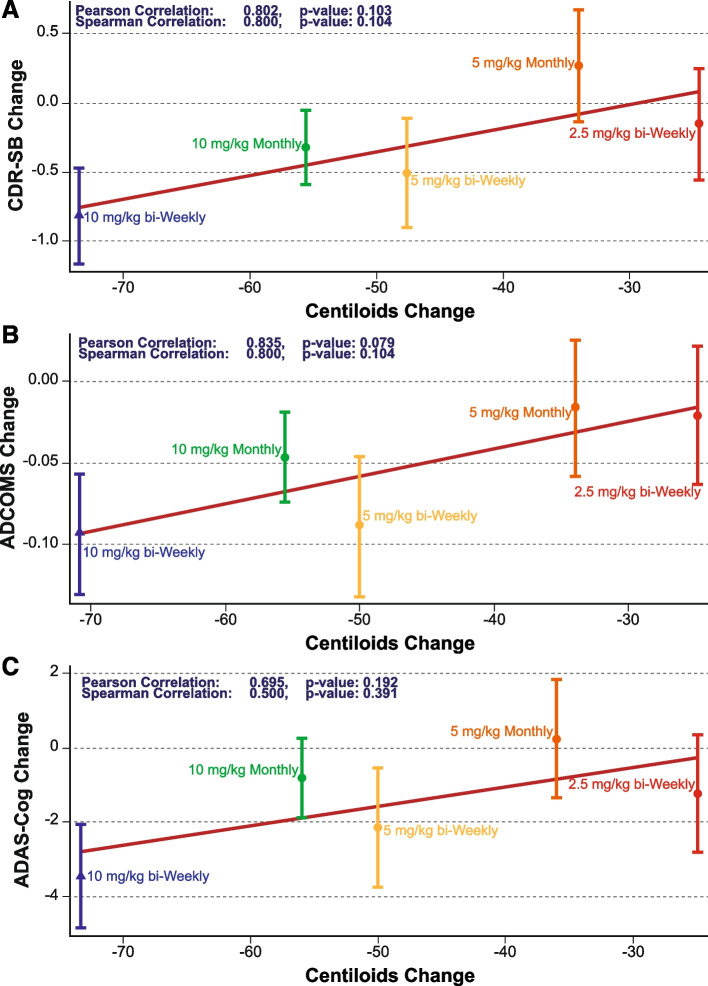
Correlation between adjusted mean differences (+/− standard error) from placebo in amyloid PET and clinical efficacy endpoints (**A** CDR-SB; **B** ADCOMS; **C** ADAS-Cog) at 18 months

The reduction in brain amyloid with lecanemab treatment as measured by amyloid PET Centiloids was associated with a slowing in clinical decline measured on the CDR-SB at the population level (Pearson correlation coefficient=0.802, P=0.103; Fig. 2) and subject level (Pearson correlation coefficient=0.119, *P*=0.059). Similar PET SUVr relationships were seen for ADCOMS at the population level (Pearson correlation coefficient=0.835, P=0.079; Fig. 2) and subject level (Pearson correlation coefficient=0.128, Pearson correlation coefficient=0.695, P=0.192) and subject level (Pearson correlation coefficient=0.057, *P*=0.373).

In the PK/PD analysis correlating lecanemab exposure with PET SUVr, ApoE4 carrier status was identified to be a significant covariate on baseline SUVr, where ApoE4 carrier subjects had higher baseline SUVr. This analysis also found that ApoE4 status had no effect on maximum plaque removal (Emax), older subjects had higher maximum plaque removal (Emax) by lecanemab, and subjects with higher baseline SUVr had greatest SUVr reduction. Change from baseline (CFB) in amyloid PET SUVr vs CFB in CDR-SB, ADCOMS, and ADAS-Cog were all successfully modeled with a linear model, and all significantly correlated, indicating reduction in PET SUVr from baseline over 18 months treatment is a significant predictor of slower disease progression (Figure S2). The effect of ApoE4 status, sex, age, and body weight were not significant in this model.

**Fig. 3:**
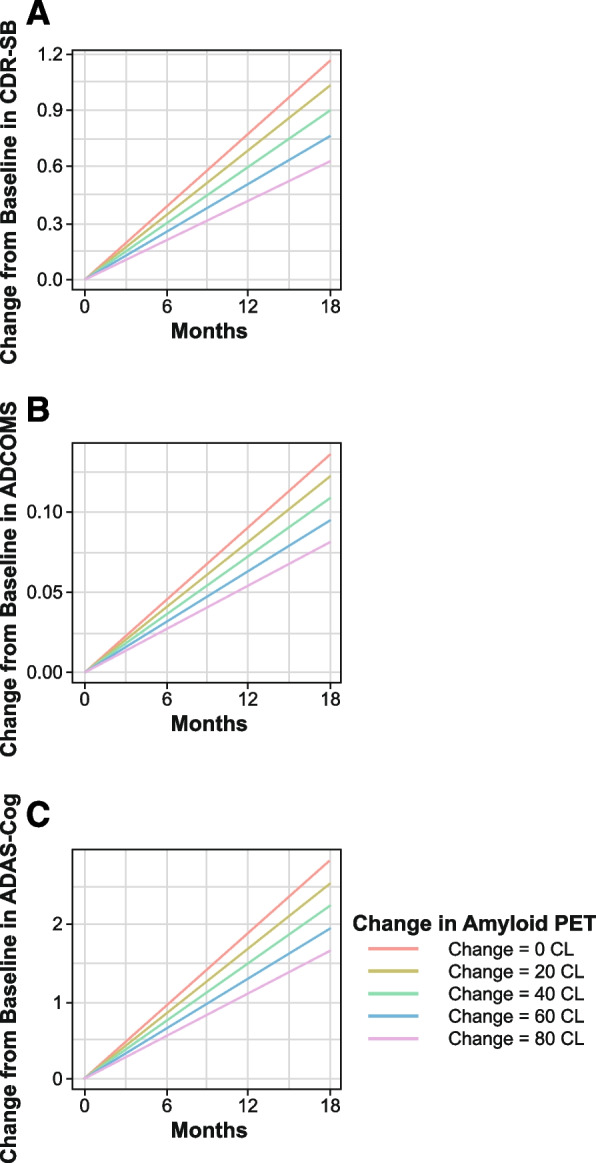
Time courses of predicted change from baseline in **A** CDR-SB, **B** ADCOMS, and **C** ADAS-Cog14 by PET SUVr change from baseline

The relationship between change of amyloid PET SUVr and clinical efficacy endpoints was explored with model-predicted CFB of PET SUVr evaluated as a predictor of the efficacy endpoints (Fig. 3). Disease progression rates for CdR-SB, ADCOMS, and ADAS-Cog14 were reduced by 11.6, 10.1, and 10.3% for every 20 CL reduction from baseline PET. Model predicted reduction from baseline PET over 18 months of lecanemab 10 mg/kg biweekly was 60.1 CL; the corresponding model-predicted reductions of disease progression rates in CDR-SB, ADCOMS, and ADAS-Cog14 were 34.8, 30.2, and 31.0%, respectively.

**Fig. 4:**
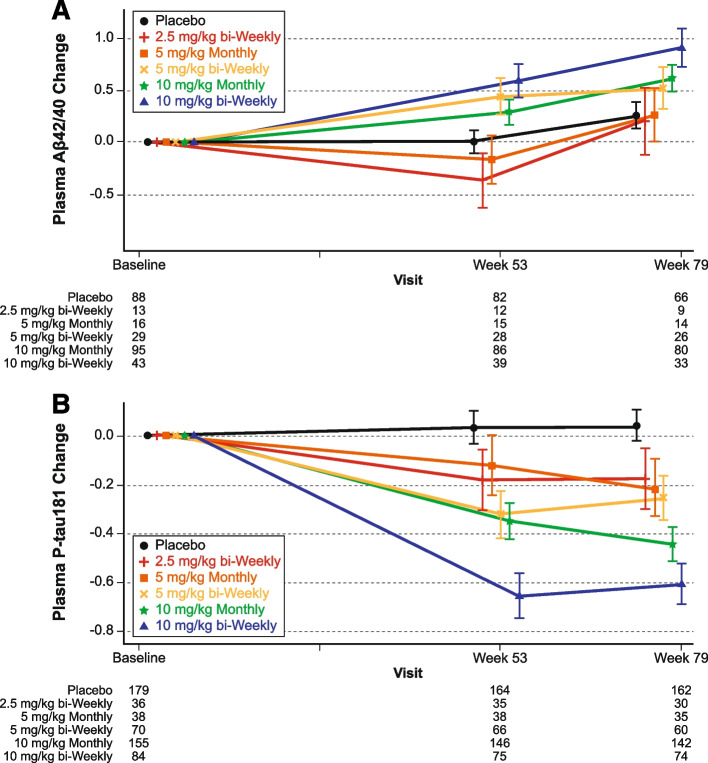
Change in **A p**lasma Aβ42/40 ratio and **B** p-tau181 by treatment group in study 201 core based on standardized value

**Fig. 5:**
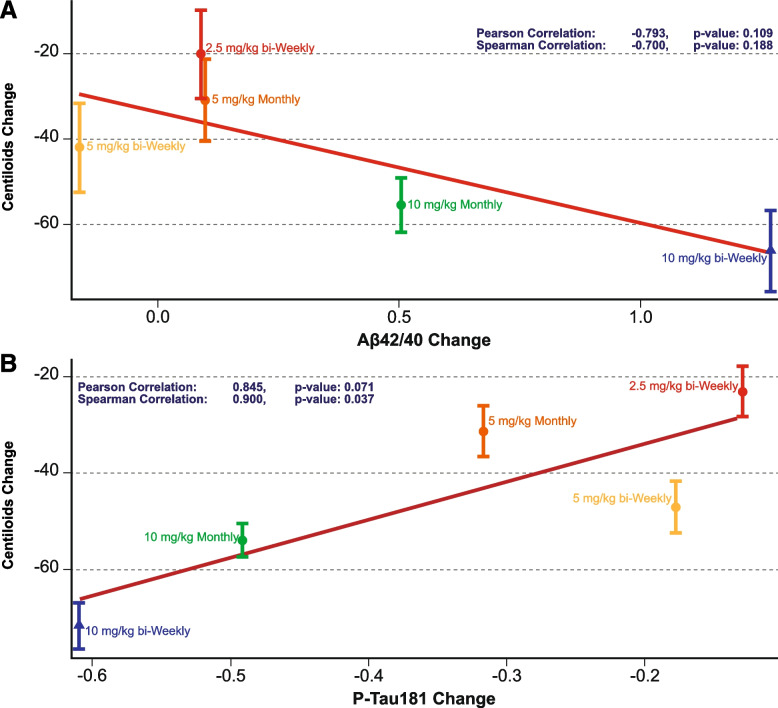
Population correlations between amyloid PET using Centiloids and plasma **A** Aβ42/40 ratio and **B** p-tau181 based on standardized value

For the plasma biomarker analysis, plasma Aβ42/40 ratio increased and plasma p-tau181 levels decreased in the combined lecanemab 10 mg/kg dose groups relative to placebo at 12 and 18 months (Fig. 4). Longitudinal changes in brain amyloid levels track with the inverse changes in plasma Aβ42/40 ratio during study 201 core. The reduction in brain amyloid with lecanemab treatment as measured by amyloid PET SUVr was associated with an increase in plasma Aβ42/40 ratio at the population level (Pearson correlation coefficient=-0.793, P=0.109; Fig. 5). Longitudinal changes in brain amyloid levels are paralleled by changes in plasma p-tau181 levels during study 201 core, with reductions in brain amyloid with lecanemab treatment associated with a decrease in plasma p-tau181 at the population level (Pearson correlation coefficient=0.845, P=0.071; Fig. 5).

**Fig. 6:**
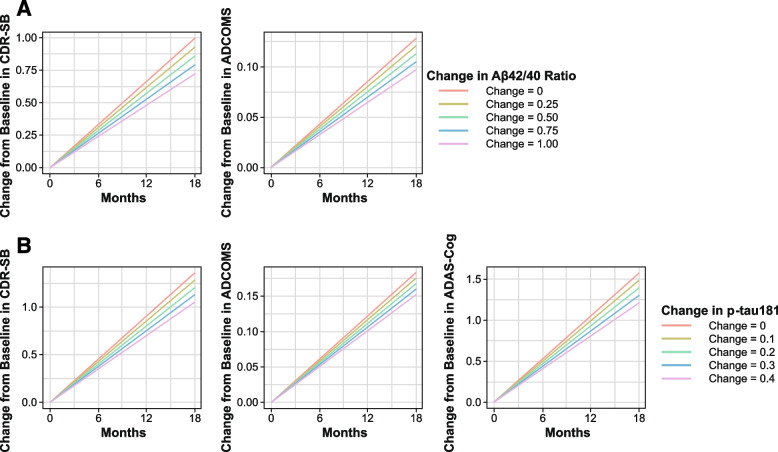
Time course of predicted change from baseline in clinical endpoints by plasma **A** Ab42/40 ratio and **B** p-tau181 change from baseline

To explore the relationship between CFB of plasma biomarkers (Aβ42/40 ratio and p-tau181) and clinical efficacy endpoints (CDR-SB, ADCOMS, and ADAS-Cog14), model-predicted CFB of the individual plasma biomarker changes were evaluated as predictors of the efficacy endpoints. For all efficacy endpoints with one exception, increase in plasma Aβ42/40 ratio or decrease in p-tau181 levels from baseline over a treatment period of 18 months was a significant predictor (*P*<0.001) of slowing of cognitive decline. The one exception was for Aβ42/40 ratio and ADAS-Cog14, where the data showed the same trend as other endpoints but did not reach statistical significance. APOE status, age, sex, and AD diagnosis (MCI, mild AD dementia) had no significant effect on relationship between plasma biomarkers (Aβ42/40 ratio, p-tau181), amyloid PET, and clinical endpoints. Time courses of predicted CFB in CDR-SB and ADCOMS by plasma Aβ42/40 ratio CFB and p-tau181 are shown in Fig. 6. Model predicted disease progression rates for CDR-SB and ADCOMS were reduced by 6.92% and 6.12%, respectively, for every 0.25 unit increase from baseline in plasma Aβ42/40 ratio. Model-predicted increase from baseline in plasma Aβ42/40 ratio over 18 months with lecanemab was 0.794 units; the corresponding model-predicted reduction of disease progression rates in CDR-SB and ADCOMS were 22.0 and 19.4%, respectively. For p-tau181, model-predicted disease progression rates for CDR-SB, ADCOMS, and ADAS-Cog14 were reduced by 5.71, 4.29 and 5.78%, respectively, for every 0.1 pg/mL reduction from baseline in plasma p-tau181. The model predicted reduction in plasma p-tau181 over 18 months with lecanemab 10mg/kg biweekly was 0.414 pg/mL; the corresponding model-predicted reduction of disease progression rates in CDR-SB, ADCOMS, and ADAS-Cog14 were 23.6, 17.7, and 23.9%, respectively.

### Gap period

**Fig. 7:**
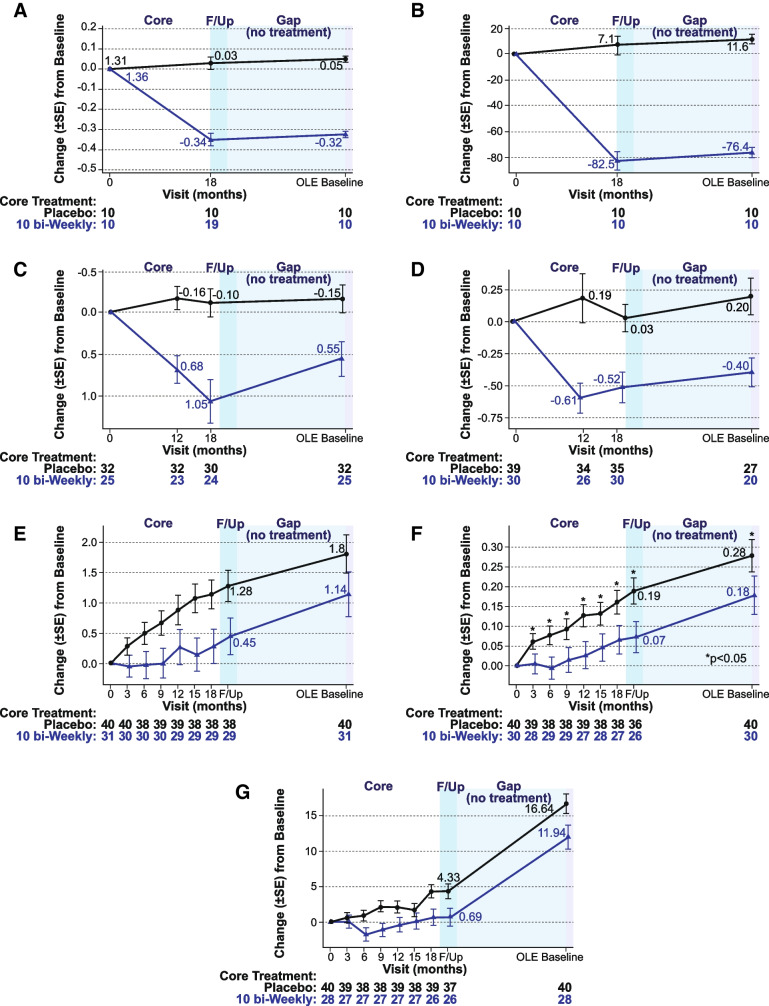
Imaging, biomarker, and cognitive effects during and after treatment with lecanemab in the study 201 core and gap period for **A** amyloid PET SUVr, **B** amyloid Centiloid, **C** plasma Aβ42/40 ratio (standardized value), **D** p-tau181 (standardized value), **E** CDR-SB, **F** ADCOMS, and **G** ADAS-cog. F/up = 90-day follow-up period following study 201 core

Lecanemab was administered for 18 months and then discontinued for an average of 24 months (range 9–59 months). During the core treatment period, substantial normalization occurred in amyloid PET, plasma Aβ42/40, and p-tau181, while the highest dose group (10 mg/kg biweekly) was partially protected from cognitive decline (i.e., ADCOMS, CDR-SB, and ADAS-Cog14) compared to placebo (Fig. 7). After the drug was stopped, lecanemab treatment differences relative to placebo observed after 18 months of treatment in subjects with global CDR 0.5 or 1 at OLE baseline were maintained on all 3 clinical assessments at a protocol-defined 3-month follow-up (off drug) visit and over the course of the off-drug gap period (OLE baseline; Fig. 7). The treated group continued to progress at the same rate as placebo over the gap period to the OLE baseline. While brain amyloid reaccumulated slightly as measured by PET during the gap period (by a mean of approximately 6 Centiloids), the soluble Aβ42/40 decreased (reaccumulated) by 47% and plasma p-tau181 levels increased (reaccumulated) by 24%. Over the gap period, the rates of clinical progression were similar between those treated with lecanemab and placebo in the core period, keeping the same absolute separation obtained at the end of treatment, but without further added benefit while off treatment.

### OLE

**Fig. 8:**
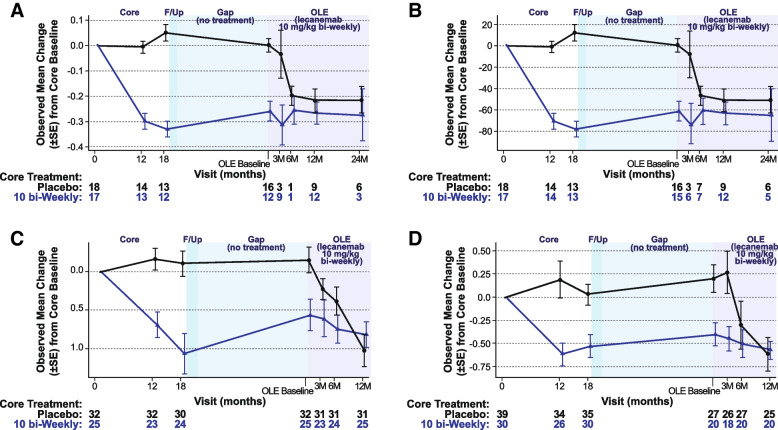
Observed mean change from baseline in **A** amyloid PET SUVr, **B** amyloid Centiloid, **C** plasma Aβ42/40 ratio (standardized value), and **D** Plasma p-tau181 (standardized value) during study 201 core, gap period, and OLE phase (OLE enrolled set). F/up = 90-day follow-up period following study 201 core

In the OLE, brain amyloid is statistically significantly reduced relative to OLE phase baseline after as little as 3 months of treatment as measured by amyloid PET SUVr and Centiloid scales, with continued brain amyloid reduction through 24 months of treatment, in subjects who received prior placebo and those who were treated with lecanemab 10 mg/kg biweekly in study 201 core (Fig. 8). In subjects on placebo in the core study who began lecanemab therapy at the start of the OLE, amyloid status by visual read converted from positive to negative in 43% (3/7) by 3 months (week 13), 75% (6/8) by the 6-month visit (week 27), 83% (10/12) by the 12-month visit (week 53) and 80% (4/5) by the 24-month visit (week 105). The findings for conversion to amyloid negative were similar when assessed by amyloid PET SUVr and Centiloid scales

**Fig. 9:**
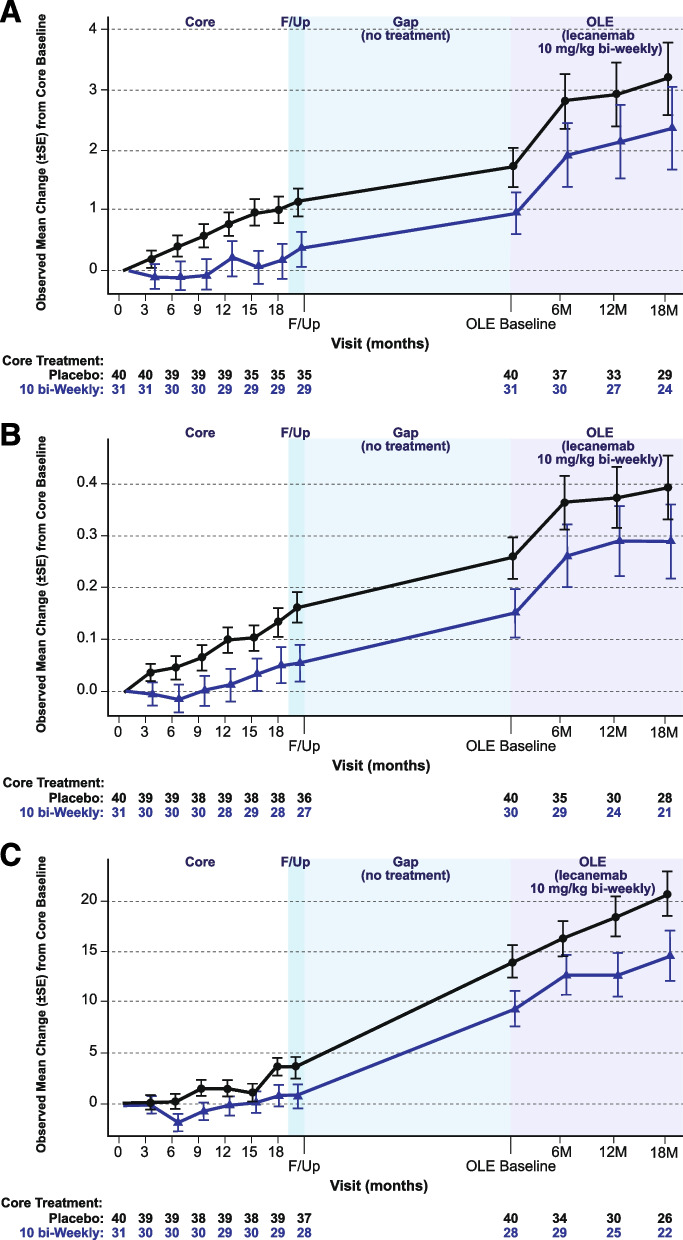
Adjusted mean change from baseline in **A** CDR-SB, **B** ADCOMS, and **C** ADAS-Cog14 during study 201 core, gap period, and OLE phase (OLE enrolled set excluding those who progressed beyond early AD). F/up = 90-day follow-up period following study 201 core

In the OLE, ADCOMS, CDR-SB, and ADAS-Cog14 scores continued to increase in both newly treated core placebo subjects and in those retreated with lecanemab (Fig. 9). Greater reduction in brain amyloid as measured by amyloid PET SUVr was associated with greater slowing of clinical decline across clinical efficacy scores. Correlation between amyloid PET SUVr and clinical assessments are provided in Figure S3.

In the OLE plasma biomarker analyses, both newly treated core placebo subjects and retreated lecanemab 10 mg/kg dosing groups showed an increase in plasma Aβ42/40 ratio and decrease in p-tau181 following treatment with lecanemab 10 mg/kg biweekly (Fig. 8). There is a strong inverse correlation between change from OLE phase baseline in plasma Aβ42/40 ratio and change from OLE phase baseline in amyloid PET SUVr at the population level (Pearson correlation coefficient=-0.744, P=0.022; Figure S4). There is a correlation between change from OLE phase baseline in plasma p-tau181 level and change from OLE phase baseline in amyloid PET SUVr at the population level (Pearson correlation coefficient=0.624, P=0.073; Figure S4). An increased reduction in brain amyloid as measured by plasma Aβ42/40 ratio was associated with greater slowing of clinical decline across clinical endpoints (Figure S5). The strongest correlation was between plasma Aβ42/40 ratio and ADAS-Cog14 (Pearson correlation coefficient=0.351; *P*=0.495); this was not statistically significant. Results for an adjusted analyses of OLE assessments can be found in the supplement (Figures S6-S8).

### Biomarker and clinical efficacy across the study 201 core, gap period, and OLE phase

To show the performance of the subset of subjects that enrolled into the OLE, we evaluated the efficacy and biomarker assessment of these subjects across the study phases. Among the subjects that participated in both study 201 core and the OLE phase, those treated with lecanemab 10 mg/kg biweekly during the core phase showed slower progression relative to placebo by the end of study 201 core, consistent with the overall core study results, although differences were not statistically significant (Fig. 9). Clinical treatment effect relative to placebo at the end of study 201 core was maintained off treatment during the gap period up to the OLE baseline, while the overall rate of progression (slope) in the gap period was similar in both groups. These differences between newly treated core placebo subjects and retreated lecanemab 10 mg/kg biweekly subjects were maintained with initiation of lecanemab 10 mg/kg biweekly in the OLE phase, although progression appeared to plateau in both groups.

Among the subjects that participated in both study 201 core and the OLE phase, change from core baseline in brain amyloid levels as measured by amyloid PET SUVr increased slightly in the placebo group during study 201 core and was markedly decreased with lecanemab 10 mg/kg biweekly, consistent with the overall core study results (Fig. 8). Amyloid PET SUVr data indicate that amyloid levels reaccumulated slightly in all subjects while off treatment over the gap period, consistent with expected natural history of amyloid accumulation rates in AD [21]. Thus, treatment discontinuation resulted in return towards pre-treatment in plasma Aβ42/40 ratio, p-tau181, and amyloid PET SUVr, recapitulating the progression of the amyloid cascade.

## Discussion

This report describes the detailed results for the lecanemab study 201 core, gap, and OLE clinical and biomarker results. In addition, the relationships between clinical measures and amyloid PET imaging and soluble biomarkers were explored with correlations analyses. Lecanemab 10 mg/kg biweekly demonstrated the largest effect among tested doses on key biomarkers and clinical endpoints, reducing brain PET amyloid (measured by visual read, PET SUVr & Centiloid scale) with corresponding changes in plasma biomarkers, while slowing of clinical decline as measured by CDR-SB, ADCOMS, and ADAS-Cog14 was observed. There were consistent parallel directional relationships between biomarker changes and changes on clinical measures. Treatment with lecanemab 10 mg/kg biweekly results in a larger and faster decrease in amyloid PET SUVr, increase in plasma Aβ42/40 ratio (a more sensitive biomarker of amyloid cascade relative to PET SUVr in this study), and decrease in plasma p-tau181 as compared to lecanemab 10 mg/kg monthly dosing.

Lecanemab concentration was a significant predictor of brain amyloid removal in PK/PD (exposure –PET) modeling expressed as a maximum effect function (Emax) [22]. Subjects with lower PET SUVr baseline achieved amyloid negativity faster than subjects with higher PET baseline values, but a higher baseline SUVr was associated with a greater magnitude of amyloid reduction. ApoE4 carrier status was identified as a significant covariate on baseline PET SUVr. ApoE4 carriers had higher baseline PET SUVr than ApoE4 noncarriers (1.39 vs 1.34). Age influenced maximum plaque removal independent of baseline PET SUVr level. For example, relative to a 72-year-old subject (median analysis set), an 84-year-old subject (95 percentile of analysis set) had 24% higher SUVr reduction, whereas a 57-year-old subject (5 percentile of analysis set) had 29% lower PET SUVr reduction. The half-life of brain amyloid re-accumulation as measured by amyloid PET was estimated to be approximately 4 years suggesting that it will take approximately 16 to 20 years (4–5 half-lives with an approximate half-life of 1.9 years for Aβ42/40 ratio degradation) for brain amyloid to reaccumulate and return to its value before lecanemab treatment.

Observed changes in plasma Aβ42/40 ratio during treatment discontinuation in the gap period suggest that stopping treatment leads to a reversal of the positive effects and at faster rate than Ab aggregation measured by PET. The plasma Aβ42/40 ratio begins decreasing again and plasma p-tau181 and amyloid PET SUVr to reverse their trajectory and start increasing, which are early indicators of brain amyloid accumulation [23, 24], associated with clinical decline during the gap period. Parallel decline in the gap period between the treated group and the placebo group may suggest that continued treatment is needed to achieve a continuing therapeutic benefit. Initiation of lecanemab in the OLE reversed these negative biomarker trends. Continued treatment with lecanemab in the OLE showed continued improvement on multiple biomarkers used to track AD processes and considered signals reflecting the biology of AD. These findings suggest that continued targeting of protofibrils with lecanemab may be beneficial for patients while still in the early AD stage, even after brain amyloid clearance as measured by amyloid PET, because other forms of amyloid may exist that are not detected by amyloid PET. Therefore, continued dosing with lecanemab to a point of normalization of the plasma Aβ42/40 and p-tau181 levels may be necessary to better determine the disease-modifying effects of normalizing Aβ.

Clinical and cognitive outcomes during the gap and OLE periods were limited by low numbers and power in this analysis, and the benefits of the drug at later stages of disease are uncertain. Data from the ongoing phase 3 CLARITY OLE study will provide a better evaluation of the effect of high-dose lecanemab on cognitive and clinical outcomes at later stages of disease.

The results presented herein lead to several noteworthy conclusions. First, rapid and thorough amyloid reduction correlates with slowing of clinical decline. Lecanemab treatment can be initiated without titration with acceptable safety (Swanson 2021). Amyloid reduction is achieved within 3 months of treatment and clinical efficacy within 6 months of treatment, with >80% of subjects amyloid negative (by visual read) by 12–18 months. Finally, there may be potential to use plasma biomarkers to monitor lecanemab treatment effects. Correlations are observed among amyloid PET SUVr, clinical endpoints, and plasma biomarkers (Aβ42/40 ratio and p-tau181) following treatment with lecanemab. Monitoring of treatment effects using plasma biomarkers may allow dose modification as needed following rapid and pronounced amyloid removal (e.g., less frequent and/or lower dose). This may obviate the need for repeat of PET scans to determine amyloid status, a current limitation in the delivery of this class of therapies to a broader population. However, the data on the effects of longer-term dosing with lecanemab on plasma biomarkers is needed to better determine the true potential of these biomarkers in monitoring therapeutic response over the long term. The more rapid return towards disease levels of plasma biomarkers relative to amyloid PET during the gap period suggests that the plasma measures may be a more dynamic measure of disease state to determine chronic dosing strategies after amyloid PET levels are normalized.

Anti-amyloid monoclonal antibodies were developed based on the central role of amyloid in AD and the hypothesis that decreasing fibrillar and protofibrillar amyloid would lead to disease modification and slowing of cognitive decline. There is not a consensus on the data needed to conclude that an agent is disease-modifying. However, three features consistent with disease modification were observed in this trial: (1) no return to the placebo level of the treated participants with cessation of therapy; (2) effects on biomarkers (Aβ, p-tau) considered important features of fundamental AD biology; and (3) persistent change in the trajectory of the illness that, generally, correlate with disease biomarkers and that supports modification of the underlying pathophysiology of the disease [25–27]. These features will contribute to the data accumulating to support the potential disease-modifying effects of lecanemab. Moreover, the temporal relationship between the soluble biomarkers and aggregated amyloid PET during the core, gap, and OLE phase provides unique information on the effects of amyloid reduction with anti-Aβ monoclonal antibodies, specifically recapitulating the sequence of events reported in observational studies. Although there have been a number of recent reports to suggest amyloid plaques are associated with the initial rise in soluble p-tau and that the reduction of amyloid plaques with anti-Aβ monoclonal antibodies results in a reduction of soluble p-tau biomarkers, the gap period in this study suggests that when anti-Aβ monoclonal antibodies are discontinued soluble amyloid (in the form of the plasma Aβ42/40 ratio) begins to return towards baseline levels, followed by plasma p-tau and both clearly precede the slow re-accumulation of amyloid PET, similar to what has been observed in observational studies [28–30]. This indicates that at the cessation of treatment, even with very low amyloid PET values, there is likely a reaccumulating of Aβ aggregates leading to the rise of both soluble Aβ and p-tau.

In addition to lecanemab, several anti-Aβ monoclonal antibodies with distinct Aβ binding profiles (e.g., aducanumab, bapineuzumab, gantenerumab, and solanezumab) have emerged and are in various stages of clinical development [27, 31, 32]. All these potential therapies are based on disease models which suggest that tau pathology is triggered by Aβ, leading to AD progression [33]. Although most previously published studies using other putative disease-modifying agents for AD did not show appreciable clinical efficacy in phase 3 [27], several recent studies have shown promising effects on reducing brain amyloid levels and slowing clinical decline [19, 34, 35]. The lecanemab mechanism of action is distinct among other anti-amyloid agents. Lecanemab has high selectivity for soluble aggregated species of Aβ compared to monomeric amyloid, with moderate selectivity over fibrillar amyloid, a profile thought to convey an advantage in selectively targeting the most toxic pathologic amyloid species [12, 15, 19].

There are several limitations of this analysis. Of the 856 randomized subjects, 180 voluntarily enrolled into the OLE. Thus, subjects were not randomized by treatment and key disease characteristics into the OLE. In addition, the OLE was started after a delay, resulting in a variable length gap period ranging from 9 to 59 months. If the OLE had started immediately after the core phase of study 201, more information on continuous dosing could have been obtained. However, the gap period presented the opportunity to observe subjects when anti-amyloid therapy was interrupted (i.e., the gap between the core and OLE) and then restarted in the OLE.

## Conclusions

In summary, an increase in exposure to lecanemab resulted in significant and clinically relevant reductions in PET SUVr and slowing of clinical decline. Our data suggest rapid and comprehensive amyloid reduction correlates with clinical benefit, potential disease-modifying effects, and the potential to use plasma biomarkers to monitor for lecanemab treatment effects. The potential for disease modification with lecanemab is supported by an increasing drug-placebo difference over time on clinical measures, a durable drug effect during the gap in dosing with the placebo group not catching up to the treatment group during the OLE, and an impact on biological measures that reflect key pathophysiological changes in AD. Clinical progression and gradual re-accumulation of pathological biomarkers supports the need for continued dosing, even after the observed clearance of brain amyloid. Our findings also suggest the potential to use plasma biomarkers to monitor for lecanemab treatment effects and potentially track individual patient responses to treatment. These results are hypothesis-generating and the potential for disease modification will be further explored in ongoing phase 3 lecanemab clinical trials in early AD and preclinical AD (Clarity AD and AHEAD 3-45, respectively).

## Study highlights

Topline efficacy and biomarker results from the randomized phase 2 lecanemab study (core) demonstrated a robust brain fibrillar amyloid reduction and slowing of clinical decline in early AD [19]. However, whether these topline efficacy and biomarker data positively correlate had not yet been addressed. In addition, OLE and gap period data that may shed additional insight into the lecanemab clinical profile had yet to be published.

We show the results from the core were maintained during a gap period and were reproduced in the OLE study. For the first time, we showed that lecanemab treatment was associated with a consistent relationship between biomarker and clinical changes and that plasma biomarkers may help guide chronic therapy. Insight from this study can be utilized to support future research and potential clinical use on dosing, monitoring of treatment effects, and the design and interpretation of future studies of anti-amyloid therapies.

## Supplementary Information

**Additional file 1.**

## Data Availability

The datasets generated during and/or analyzed during the current study are available from the corresponding author on reasonable request.
