# Supplementary material for: Lecanemab in patients with early Alzheimer’s disease: detailed results on biomarker, cognitive, and clinical effects from the randomized and open-label extension of the phase 2 proof-of-concept study
Source: Alzheimers Res Ther. 2022 Dec 21;14:191. doi: 10.1186/s13195-022-01124-2 (PMC9768996; doi:10.1186/s13195-022-01124-2)
Supplement: Supplementary file 1 — Additional file 1. [file 13195_2022_1124_MOESM1_ESM.docx]

**Supplemental Appendix**

**Drug interference with plasma amyloid 42/40 and ptau181 assays:**

Lecanemab drug interference for plasma p-tau181 has been conducted and no interference was observed for the in human plasma in the presence of 2100 µg/mL and 600 µg/mL of BAN2401. No drug interference was observed for measuring plasma Aβ40, Aβ42 and Aβ42/20 in the presence of lecanemab 260pg/mL and 780pg/mL. Additional drug interference assessments have been planned with higher concentrations (drug level 260 ug/mL, 600ug/mL and 2100 ug/mL).

**Supplemental Tables and Figures**

**Figure S1.** Lecanemab Study 201 Study Design

Aβ, amyloid-beta; AD, Alzheimer’s disease; ADAS-cog, Alzheimer’s Disease Assessment Scale-cognitive subscale; ADCOMS, Alzheimer’s Disease Composite Score; CDR, clinical dementia rating; CDR-SB, Clinical Dementia Rating Scale sum of boxes; IV, intravenous; MCI, mild cognitive impairment; OLE, open label extension; PET, positron emission tomography.

*Any subject who completed study treatment (Visit 42 [Week 79] of the Core Study) and fulfilled the Extension Phase eligibility criteria had the option to participate in the Extension Phase. Subjects who previously completed the Core Study (through the Follow-Up Visit, Visit 43) at any time before implementation of the Extension Phase and/or fulfilled the Extension Phase eligibility criteria were eligible to participate. Any subject who had discontinued the Core Study but fulfilled the Extension Phase eligibility criteria were eligible to participate in the Extension Phase (subjects who discontinued due to ARIA were allowed to enroll if they were otherwise eligible). All subjects had a Follow-Up Visit 3 months after the last dose of study drug in the Core. ^†^Randomization assignment was determined by Bayesian Adaptive Design methodology.

**Figure S2.** Change from Baseline (CFB) in Amyloid PET using Centiloids vs. CFB at 18 and 12 months in (A) ADCOMS, (B) CDR-SB, and (C) ADAS-cog14 Modelled with a Linear Model

**A. CDR-SB**

WCM = whole cerebellum mask.

**B. ADCOMS**

**C. ADAS-cog14**

**Figure S3.** Correlation Between Amyloid PET using Centiloids and Clinical Endpoints (A. CDR-SB, B. ADCOMS, C. ADAS-Cog) during OLE

**A. CDR-SB**

**
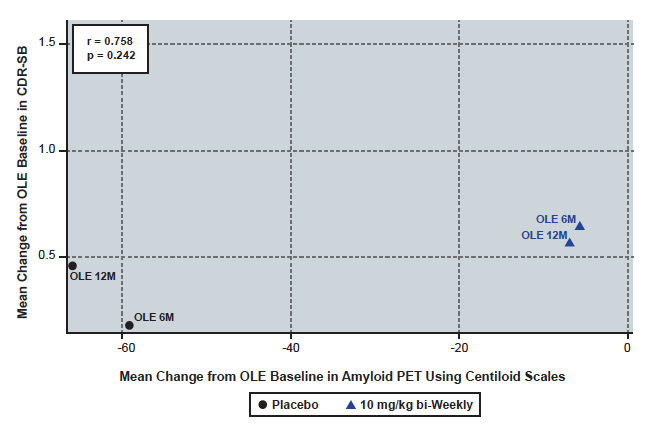
**

**B. ADCOMS**

**
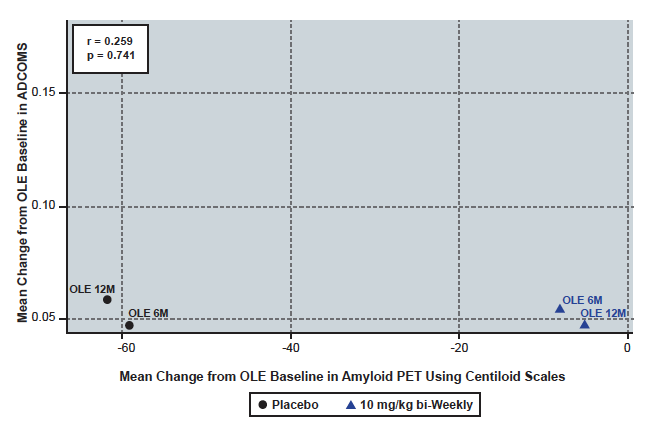
**

**C. ADAS-Cog**

**
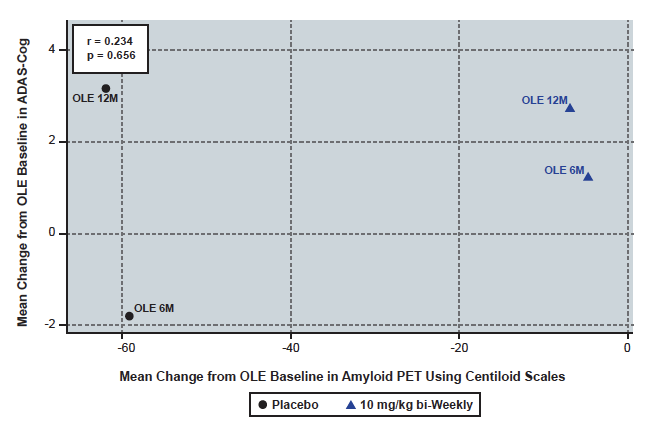
**

**Figure S4.** Correlation Between Amyloid PET using Centiloids and Biomarkers (A. Aβ42/40, B. p-tau181) during OLE based on standardized value

**A. Aβ42/40**

**
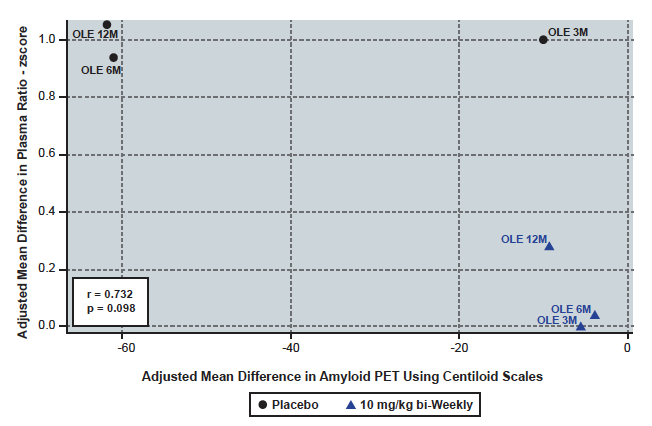
**

**B. p-tau181**

**
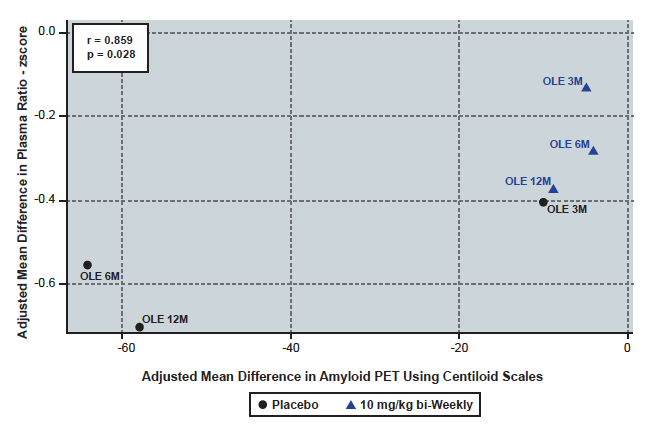
**

**Figure S5.** Correlation Between Plasma Aβ42/40 ratio based on standardized value and Clinical Endpoints (A. CDR-SB, B. ADCOMS, C. ADAS-Cog) during OLE
**A. CDR-SB**

**
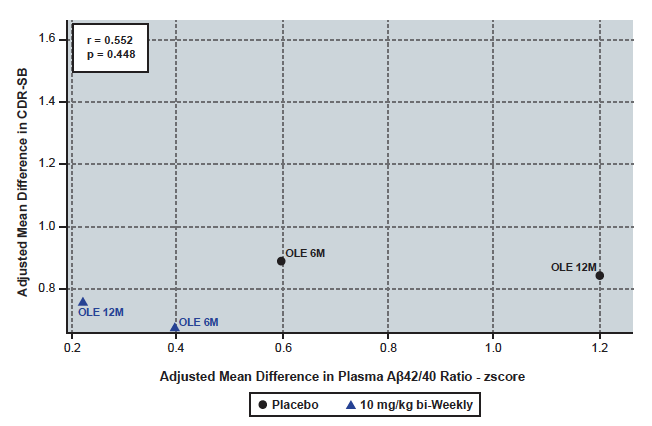
**

**B. ADCOMS**

**
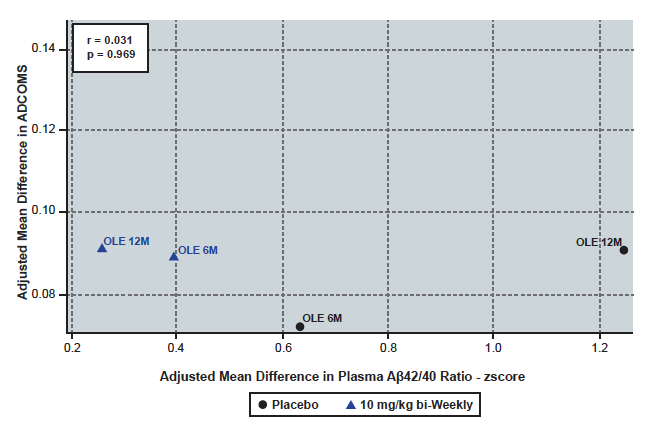
**

**C. ADAS-Cog**

**
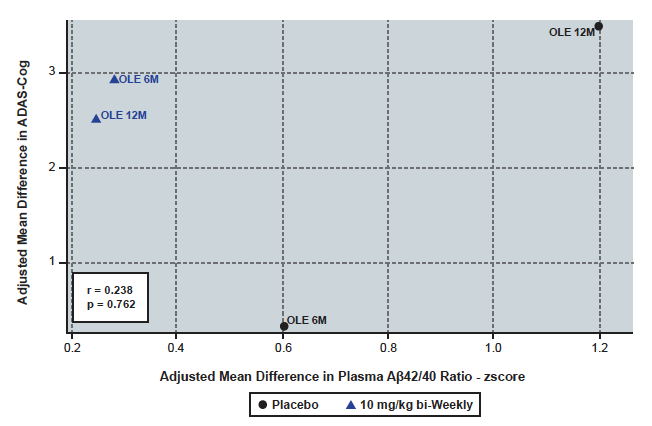
**

**Figure S6.** Study 201 OLE-Results for (A) Amyloid PET SUVR and (B) Centiloid Scales for Subjects who were Previously Untreated (Placebo) and Previously Treated with 10 mg/kg Bi-weekly Lecanemab

1. **PET SUVr**

**
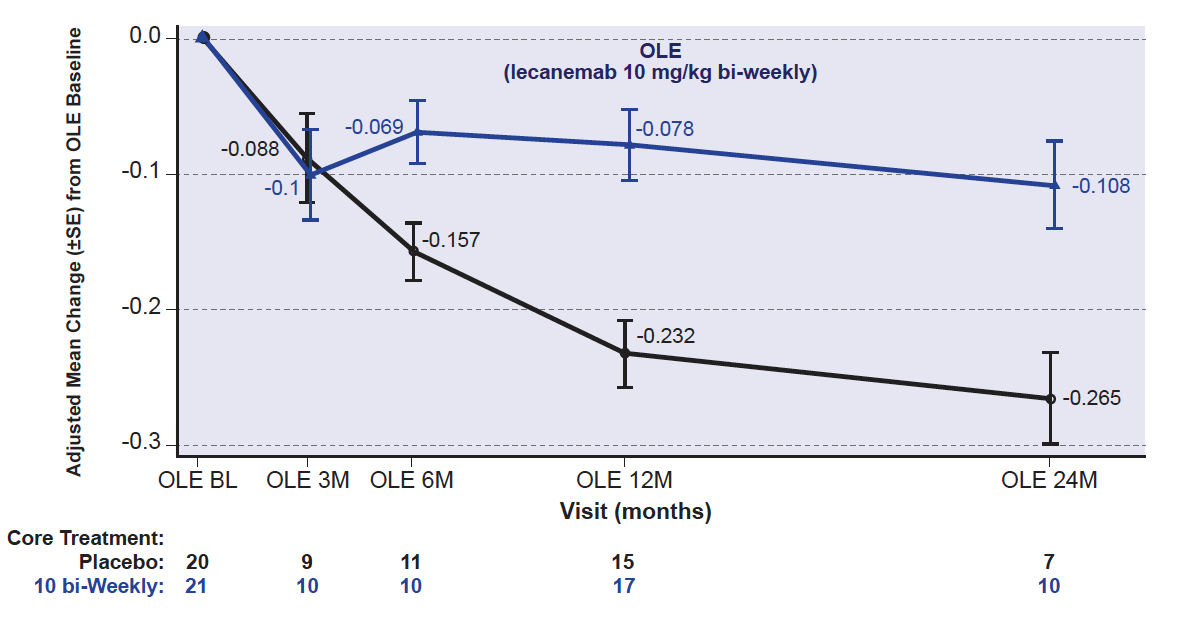
**

1. **Centiloid Scales**

**
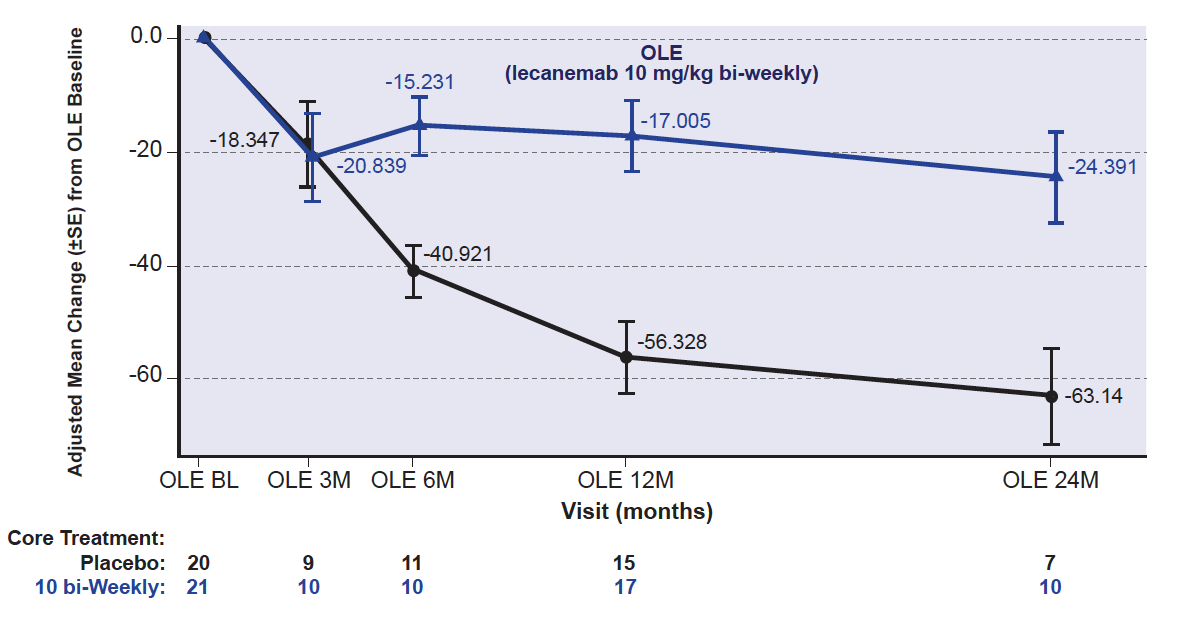
**

**Figure S7.** Mean Change (SE) From OLE Baseline in (A) CDR-SB, (B) ADCOMS, and (C) ADAS-cog14 in the Study 201 OLE Phase for Subjects who were Previously Untreated (Placebo) and Previously Treated with 10 mg/kg Bi-weekly Lecanemab

**A. CDR-SB**

**
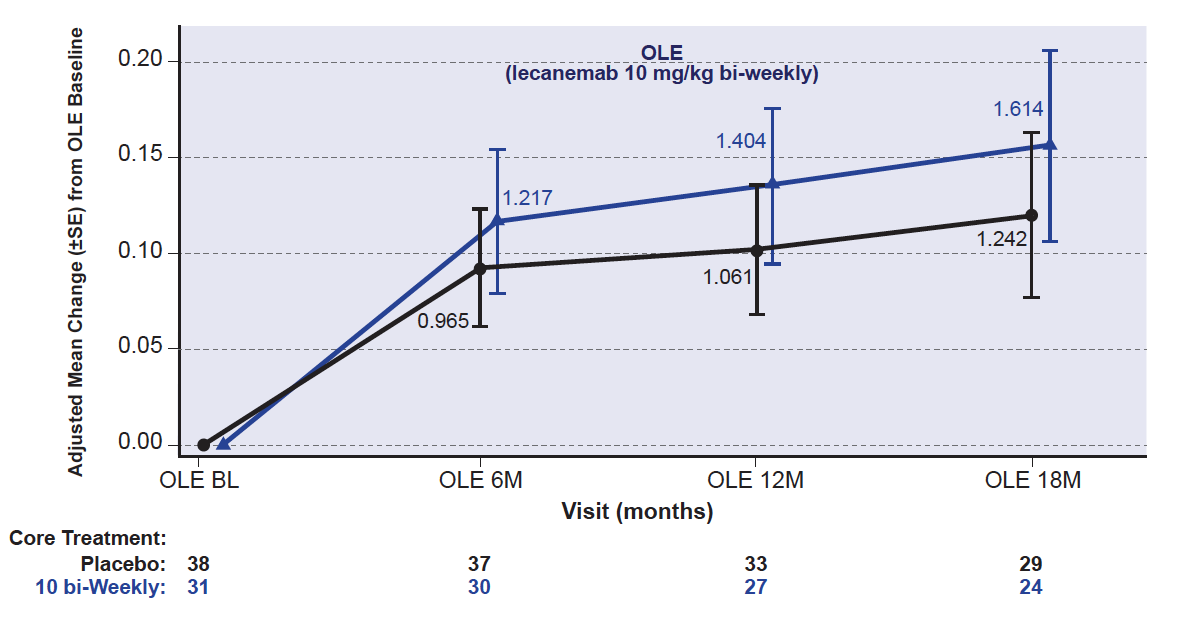
**

**B. ADCOMS**

**
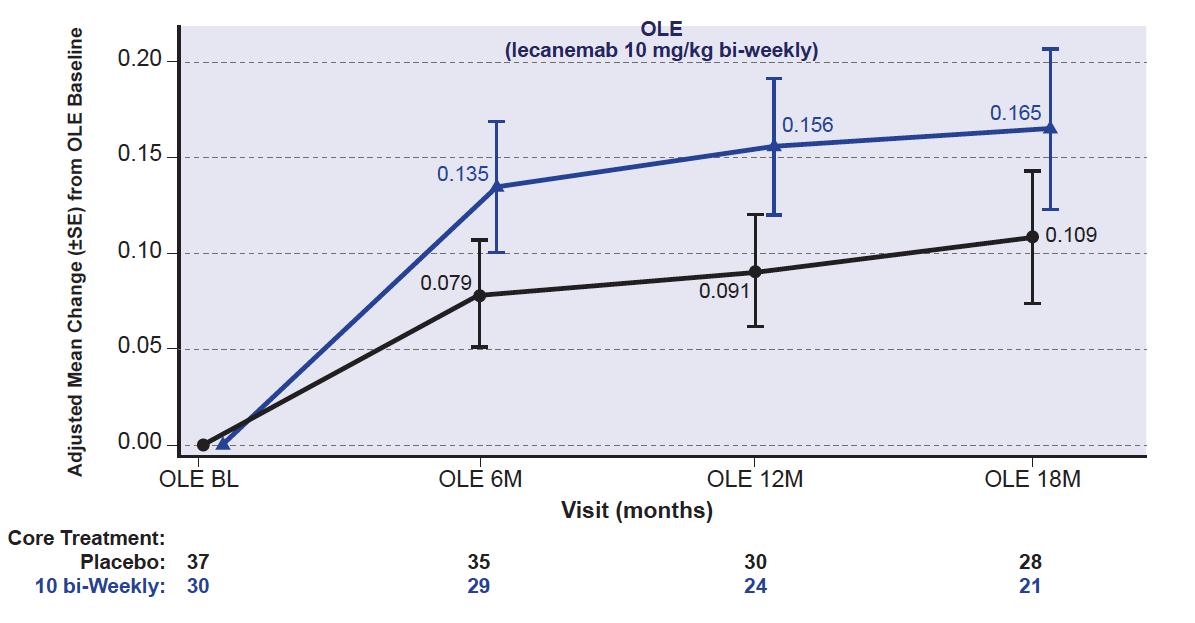
**

**C. ADAS-cog14**

**
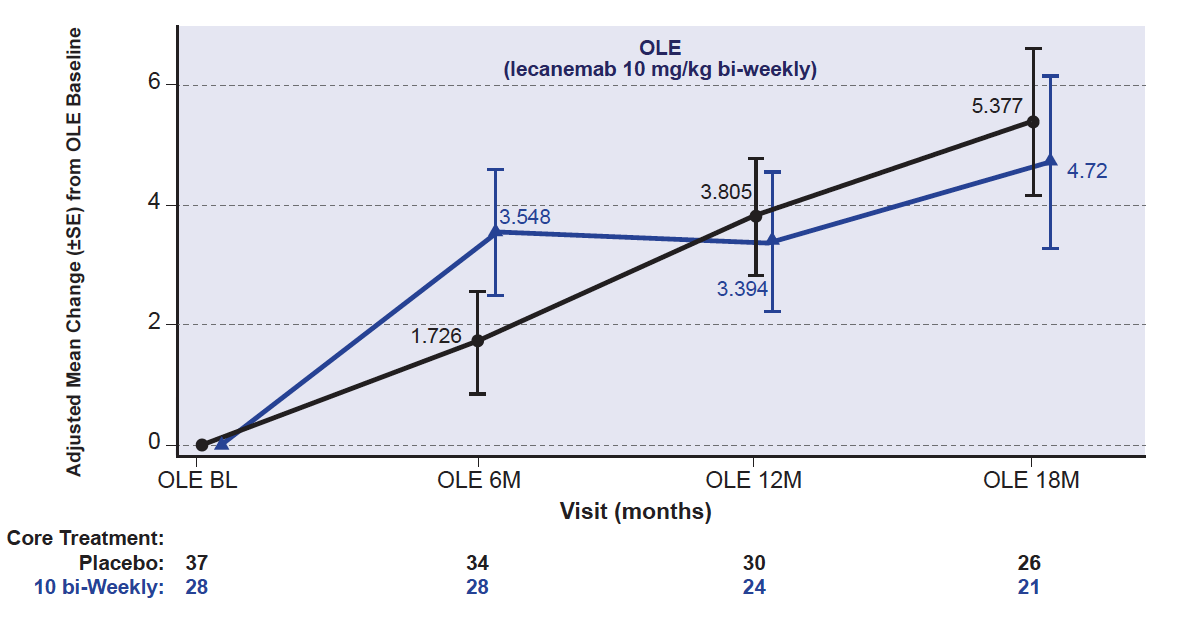
**

**Figure S8.** Mean Change (SE) in (A) Plasma Aβ42/40 Ratio and (B) p-tau181 based on standardized value by Visit in the Study 201 OLE Phase for Subjects who were Previously Untreated (Placebo) and Previously Treated with 10 mg/kg Bi-weekly Lecanemab

**A. Plasma Aβ42/40 Ratio**

**
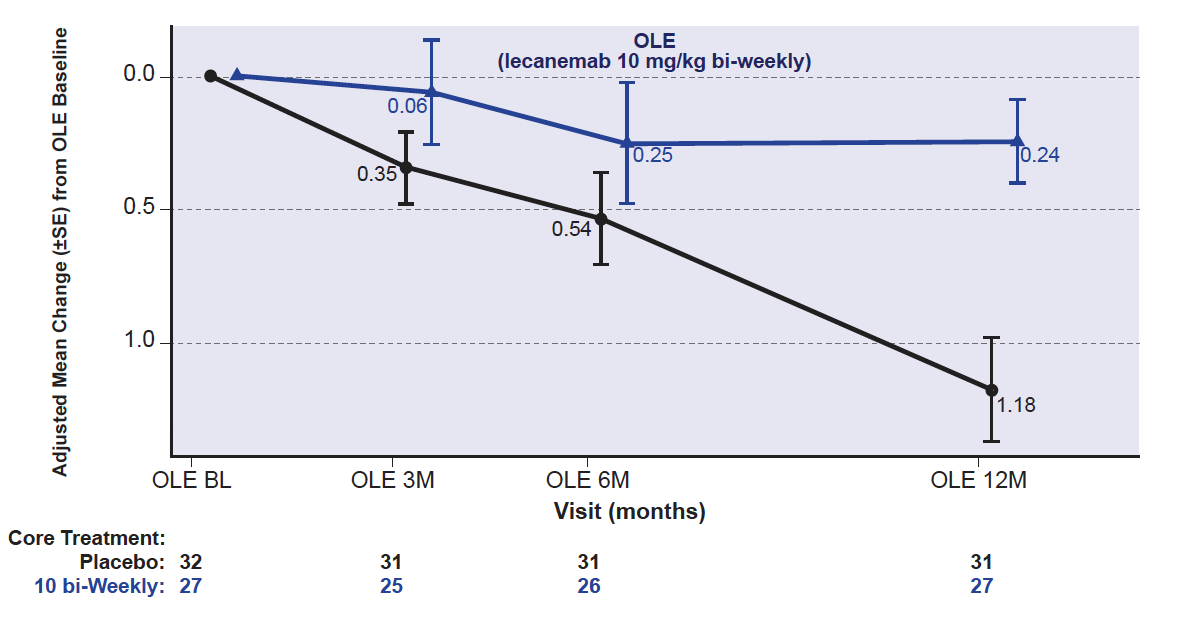
**

**B. p-tau181**

**
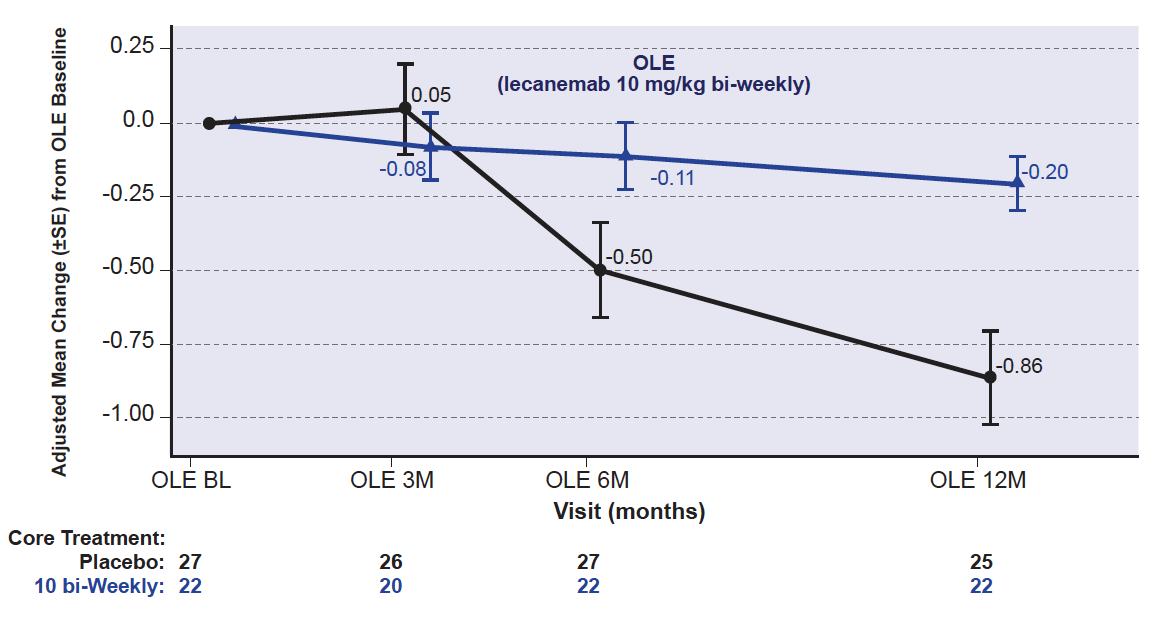
**
